# Investigation of nutritional properties of three species of marine turban snails for human consumption

**DOI:** 10.1002/fsn3.360

**Published:** 2016-04-05

**Authors:** Roslizawati Ab Lah, Joshua Smith, Dale Savins, Ashley Dowell, Daniel Bucher, Kirsten Benkendorff

**Affiliations:** ^1^ Marine Ecology Research Centre, School of Environment, Science and EngineeringUniversity of Southern CrossLismore2480New South WalesAustralia; ^2^ University Malaysia TerengganuKuala21030TerengganuMalaysia; ^3^ Southern Cross Plant ScienceUniversity of Southern CrossLismore2480New South WalesAustralia

**Keywords:** Fatty acids, heavy metals, *n*−3/*n*−6 ratio, protein, trace elements, turban snails, Turbinidae

## Abstract

Turban snails (family Turbinidae) are gastropod molluscs that are harvested for human consumption yet little is known about the nutritional properties of these snails, particularly from Australian waters. This study compares the proximate composition (ash, moisture, protein, and lipid content), fatty acid profiles, mineral, and trace element content of three species of turbinid snails; *Turbo militaris, Lunella undulata,* and *Lunella torquata* from northern New South Wales, Australia. They were all found to have relatively high protein in their flesh (16.0% to 18.5% of the fresh weight). *L. torquata* had a significantly higher lipid content (8.5% w/w) than *L. undulata* (5.2% w/w), whereas *T. militaris* (5.6% w/w) was not significantly different to either. Analysis with gas chromatography showed there was no significant difference in monounsaturated fatty acid (MUFA) content, with an average of approximately 14% of the total fatty acids in all three species. However, saturated fatty acids (SFA) were significantly higher in *T. militaris* (41%), whereas polyunsaturated fatty acids (PUFA) were significantly higher in *L. undulata* (46%). The ratio of *n*−3/*n*−6 fatty acids ranged from 1.1 in *T. militaris* to 1.4 in *L. torquata,* which is good for human health and comparable to other high value gastropods. The results indicate that *T. militaris*,* L. undulata,* and *L. torquata* provide a good source of essential elements such as zinc, selenium, and iron. At the location studied, toxic metals and metalloids were below safe recommended standards for human consumption. Overall, this study confirms the suitability of turban snails as a nutritional food for human consumption.

## Introduction

Many marine molluscs are harvested around the world for their meat. They are important resources that contribute considerable economic value to the world's fisheries (Leiva and Castilla 2002). In the year 2013, the commercial harvest of at least 9.8 million tons of molluscs was reported as part of the world fisheries catch (FAO, 2015a). The majority of the molluscan fishery is contributed by bivalves and cephalopods, with gastropods contributing less than 2% of the total harvest (FAO, 2015b), although some gastropods do have a relatively high economic value (Leiva and Castilla 2002). The demand for global fisheries product, including molluscs, increases every year as the human population grows (Naylor et al. 2000; Diana 2009) leading to exploitation of new stocks (Dey 2015). Consequently, there is a need for supplementing the current mollusc catch with new or underutilized species.

Molluscs have been recognized as a high‐quality nutritious food source and many species are considered as culinary delicacies. A considerable amount of literature has been published on the nutritional composition of some molluscs. For example, several studies have reported relatively high protein levels found in mollusc flesh, including abalone *Haliotis diversicolor* (Chiou et al. 2001) and oysters *Crassostrea gigas* (Linehan et al. 1999; Dridi et al. 2007). The nutritional quality of mollusc flesh lies not only with the high quality of protein, but also in its relatively low lipid content and high proportion of polyunsaturated fatty acids (PUFAs) (Nichols et al. 1998; Mooney et al. 2002).

Molluscs are known to contain a wide variety of PUFAs, some of which are considered as essential fatty acids that humans cannot synthesize and must be obtained from food (Smoothey 2013). Among the PUFA's, long‐chain omega‐3 fatty acids, such as eicosapentaenoic (EPA), docosapentaenoic (DPA), and docosahexanoic (DHA), are thought to play beneficial roles in a healthful diet. The balanced intake of omega‐3 (*n*−3) and omega‐6 (*n*−6) fatty acid can help prevent cardiovascular disease (Mahaffey 2004; Mahaffey et al. 2008), coronary heart disease, arthritis (Simopoulos 1991), and other inflammation (Milinsk et al. 2003; Mahaffey 2004; Mahaffey et al. 2008). Since these *n*−3 and *n*−6 fatty acids cannot be synthesized by molluscs, they must be acquired from their food intake, such as phytoplankton or algae (Foster and Hodgson 1998). As a consequence, the fatty acid content is expected to vary within and between mollusc species depending on their specific diets and over time as the populations of algal species fluctuate. Physiological demand for certain fatty acids during the reproductive cycle could also contribute to temporal variation and differences between the sexes (Brazao et al. 2003).

The nutritional content and fatty acid compositions of molluscs has been intensively investigated in commercially important bivalve species, including oysters (Ostreidae) (Saito and Hashimoto 2010), scallops (Pectinidae) (Napolitano and Ackman 1992; Pazos et al. 1997a,b), pearl oyster (Pteriidae) (Saito 2004; Gokoglu et al. 2006), and mussels (Mytilidae) (Chan et al. 2004; Su et al. 2006). Nevertheless, there is less information available on the biochemical components of other molluscs. In particular, only a few studies have been published on fatty acid composition and nutritional quality of the flesh of gastropods, including members of the families Haliotidae (Dunstan et al. 1996; Chiou et al. 2001; Nelson et al. 2002; Su et al. 2006), Turbinidae (Freiji and Awadh 2010; Nooshin and Peyman 2011; Saito and Aono 2014), Babyloniidae (Periyasamy et al. 2011), and Muricidae (Woodcock and Benkendorff 2008; Vasconcelos et al. 2009).

Molluscs are also known to be among the best bio‐indicators for monitoring environmental pollution in coastal waters, due to their relative immobility and their ability to accumulate high concentrations of heavy metals from the surrounding environment (Metian et al. 2008; Jakimska et al. 2011). Increasing urbanization and industrialization could potentially increase the contamination of marine ecosystems through discharge of sewage, industrial runoff, and agricultural waste (Chouvelon et al. 2009; Pan and Wang 2012). The capacity to accumulate metals may vary between species and individuals depending on size or physiological condition (Cubadda et al. 2001; Duquesne et al. 2004; Mubiana et al. 2006; Bille et al. 2015). If the concentrations in molluscs exceed the permitted concentration, this may pose a health risk to humans.

A study on the turbinid gastropod *Cookia sulcata* highlighted the presence of essential dietary minerals such as zinc and other nutritional components (Mason et al. 2014). Several studies have been undertaken on the nutritional and elemental composition of other gastropods, such as the muricids *Chicoreus ramosus* (Xavier Ramesh and Ayyakkannu 1992), *Rapona venosa* (Celik et al. 2014) and the babyloniid *Babylonia spirata* (Periyasamy et al. 2011), as well as bivalves such as the oysters *Crassostrea rhizophorae* and *Ostra edulis* (Karakoltsidis and Zotos 1995; Martino and Cruz 2004), the venerid clams *Meretrix casta* and *Protothaca thaca* (Olmedo et al. 2013; Smoothey 2013), the mussel *Mytilus galloprovincialis* (Karakoltsidis and Zotos 1995; Olmedo et al. 2013) and the cockle*, Ostra edulis* (Karakoltsidis and Zotos 1995) and *Mytilus galloprovincialis*,* Cerastoderma edule* (Olmedo et al. 2013).

**Figure 1:**
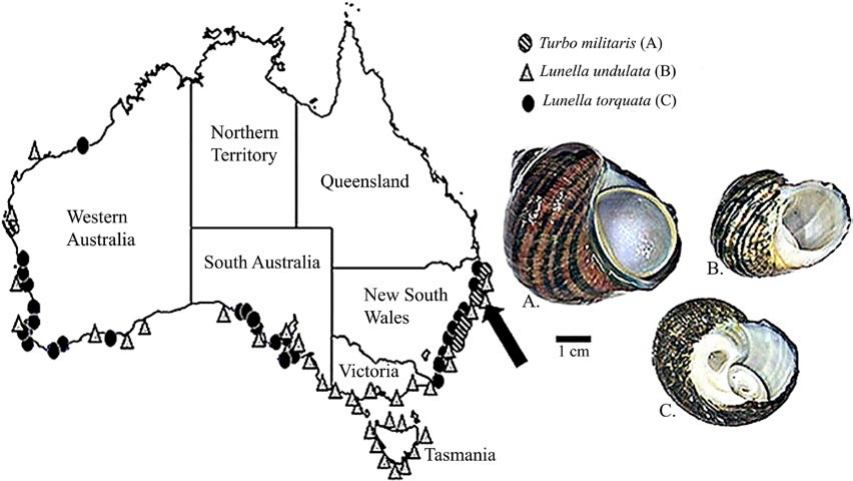
Map showing the distribution of the three turban snails: A) *Turbo militaris* Reeve, 1848; B) *Lunella undulata* Lightfoot, 1786; C) *Lunella (Ninella) torquata* Gmelin, 1791, in Australia with the arrow indicating the location of the sampling site (Woolgoolga NSW; ‐30.108300, 153.207003). Adapted from: (Bouchet 2015), Australian Faunal Directory (accessed Oct 2015).

Turban snails are abundant in most coastal seas (Joll 1980; Smoothey 2013; Saito and Aono 2014). In Japan, Korea, and China, turban snails are highly valued as a food (Chen et al. 2004; Mason et al. 2014) and have been described as being “among the best gastropods for human consumption” by Yearsley et al. (1999). The turban snail fishery in Australia is currently a small‐scale fishery with annual commercial landings of approximately 7 tons in New South Wales and approximately 6.5 tons in South Australia (PIRSA, 2010; Rodellar et al. 2010). However, the Turbinidae can also be subject to substantial recreational harvest (Cooling and Smith 2015). Three species in family of Turbinidae that commonly occur in temperate‐to‐subtropical waters of Australia are *Turbo militaris*,* Lunella torquata,* and *L. undulata*. They can be found in shallow rocky reef habitats along the eastern and southern coasts of Australia (Fig. 1). The distributions of all three species overlap in the subtropical waters of northern New South Wales (NSW). This provides an opportunity to directly compare the nutritional properties between these species without confounding effects from different locations. No previous studies have been undertaken to investigate the nutritional properties of these Australian Turbinidae species. Therefore, the aim of this study was to assess the nutritional content of *T. militaris*,* L. undulata,* and *L. torquata*, from NSW, Australia, specifically the proximate composition (i.e., protein, lipid, carbohydrate, moisture, and ash), fatty acid profiles and micronutrient and trace elemental concentrations.

## Materials and Methods

### Collection and preparation of specimens

The snails were all collected from Woolgoolga, Coffs Harbour, Australia (Fig. 1) under fisheries permit (permit number: P13/0002–1.0) in May 2014. To eliminate the effects of variations in trace elements associated with different localities and temporal variations in proximate composition due to the reproductive cycle and seasonal variations in food supply, three species were collected by hand from the same locality on the same low tides. In total, six specimens of *L. undulata*, four specimens of *T. militaris,* and four specimens of *L. torquata* were collected. All the specimens were transported to the laboratory in a 10 L bucket and processed within 4 h of collection. The shell was broken using a bench vice and the foot muscle and viscera removed. Foot tissue was used for all analyses, as this is the part of the animal used for human consumption. Each specimen was divided into four pieces weighing approximately 1 g each. All tissue weights were recorded for the proximate composition analysis using an analytical balance with precision of ± 0.0001 g (model ML 204/01; Mettler Toledo, Plainview, NY). One portion of each snail was used for moisture and ash analysis, another for lipid and fatty acid analysis, a third for protein analysis and the forth for elemental composition. Portions from each snail were analyzed separately to provide four to six replicate analyses per species.

### Nutritional proximate analysis

All reagents and chemicals in this study were purchased from Sigma (St Louis, Mo). Moisture content was calculated based on the percentage weight loss after drying to a constant weight at 60°C for 24–48 h (Woodcock and Benkendorff 2008). To determine the ash content, the dry samples were weighed and transferred to a muffle furnace (model KSL 1700X; MTI Corporation, Richmond, CA) at 550°C for 4 h.

Lipids were extracted from a second piece of tissue from each specimen according to the methods of Woodcock and Benkendorff (2008) based on the original method by Bligh and Dyer (1959). Samples were immersed for 1.5 h in the solvent (1:2 chloroform methanol) (v/v) then filtered through Whatman #1 filter paper (Sigma‐Aldrich; Munich, Germany) into a preweighed clean test tube (culture test tube with Teflon lined screw cap). Solvent replacements continued until no further color was extracted from the tissue. All the solvent fractions were then combined and the extracts were dried under a stream of high‐purity nitrogen gas until no solvent remained, then reweighed.

### Analysis of fatty acid composition

The lipid extracts were subject to fatty acid analysis by gas chromatography after derivatization into fatty acid methyl esters (FAMEs). To prepare the methyl esters, 1.5 mL of a 0.5 mol/L solution of sodium hydroxide in methanol was added to the lipid extracts (Kanthilatha et al. 2014). The samples were heated in a dry block at 100°C for 10 min. To complete the methylation of fatty acids, 2 mL of boron trifluoride in methanol was added and heated at 100°C for a further 30 min. The samples were then cooled to room temperature before 1 mL of hexane was added to extract the fatty acid methyl esters. The tube was shaken vigorously for at least 30 sec and 5 mL of saturated sodium chloride solution was added followed by shaking thoroughly for at least 5 sec. The polar and lipophilic solvent layers were allowed to separate and the upper hexane layer transferred to an autosampler vial for gas chromatography (GC) injection.

The composition of fatty acids was determined using standard FAMEs analysis by gas chromatography. The gas chromatograph used was an Agilent 6890 N, equipped with a FID (flame ionization detector). The Agilent 6890 split injector and FID were maintained at 230°C and 260°C, respectively. The capillary column used was BPX 70 (70% cyanopropyl polysilphenylene‐siloxane, 50 m × 0.22 mm × 0.25 *μ*m). The GC oven temperature was programmed with 100°C hold for 5 min and then increased at a rate of 5°C/ min until the final temperature 240°C was reached. The carrier gas was Helium. One microliter of the extract containing the fatty acids methyl esters was injected with a split ratio of 200:1, and a column flow of 1 mL/min. The retention time of each peak detected in the samples was compared to the FAMEs test mix (Sigma) and the area under the curve was calculated to determine the percent composition relative to all peaks.

Some supplementary analyses were undertaken using gas chromatography/mass spectrometry (GCMS) to confirm the identity of all fatty acid methyl esters and identify the unknown peaks that did not correspond to the FAMEs standards. The GCMS (Agilent 6890) was coupled with an Agilent 5973 mass selective detector. The mass spectra were recorded at 70 eV ionization voltage over the mass range 35–550 amu. The identification of unknown peaks was based on matching to a mass spectral library (WILEY 275 and NIST98).

### Protein analysis

For protein determination, 1 g of foot tissue was digested in 10 mL of 1 mol/L sodium hydroxide (NaOH) until all tissue completely dissolved. After digestion 1 mL of sample was transferred to an Eppendorf tube and centrifuged in a rotary microfuge for 5 min at 96.15 g. Bovine serum albumin (BSA) was used as a standard protein solution (10 mg/mL) and a twofold dilution series was created (10.000–0.0195 mg/mL). The BSA standard protein solutions and all the digested snail protein samples were subjected to the Biuret Assay (Brooks et al. 1995). For a negative control, 1 mol/L NaOH was used. The absorbance of protein in the samples was measured at 550 nm on a spectrophotometer (model Victor X4; Perkin Elmer, Waltham, MA). The shift at 550 nm results from the reagents reacting with the peptide bonds of proteins, with absorbance intensity directly proportional to the concentration of protein (Okutucu et al. 2007). The concentration of all the unknown samples was determined using the linear regression equation from absorbance values generated by the BSA standard curve.

### Analysis of mineral concentrations

For mineral analysis, 1 g samples of foot tissue were submitted to the Environmental Analysis Laboratory (EAL), Southern Cross University (NATA Accreditation Number 14,960). Samples of foot tissue were dissolved in a mixture of HNO_3_ (25%) and HCL (75%) (1:3, v/v) and subjected to hot‐block (Hot‐Block; Environmental Express, Charleston, South Carolina) acid digestion procedure (APHA, 2012). Mineral concentrations were analyzed by inductively coupled plasma mass spectrometry (ICPMS) using a NexION 300 D series ICP spectrometer with an ESI SC‐FAST Auto Sampler (Perkin Elmer, Waltham, MA).

### Statistical analysis

The data are expressed as means ± standard error. For parametric univariate analyses, homogeneity of the data was explored with Levene's test. Log transformation was applied if the residuals of the data in the ANOVA model were not normally distributed (Kolmogorov–Smirnoff test). Data with homogeneous variances were analyzed using Analysis of Variance (ANOVA) and *post hoc* Tukey's LSD multiple comparisons to determine differences between species. ANOVA tests were performed using the software package SPSS for windows, version 20 (IBM Corp., Armonk, New York). Multivariate data and data that could not be transformed to meet the assumptions of ANOVA were tested using permutational analyses in PRIMER v 6 +  PERMANOVA add‐on (v.6, PRIMERe, Pty. Ltd., Plymouth, UK). Euclidean distance similarity matrices were created from the percentage composition data for fatty acids or after normalization to the same scale for the mineral and trace element concentration analysis. One‐way PERMANOVAs were run using 9999 permutations of the data to determine overall differences between species. When there was a significant species effect, post hoc pairwise tests were used to determine which pairs of species differed. Principle component ordination (PCO) was also undertaken to visually display the differences between species in fatty acids and trace elements, with vector overlay using Pearson correlation >0.8 and >0.5, respectively. In all analyses, a significance of *α * =  0.05 was used.

## Results

### Proximate analysis

The proximate compositions of the three turban snail species are reported on a fresh weight basis in Table 1. No significant difference (*P *<* *0.05) in the ash content was observed between the three different species, with an average around 2% w/w. The moisture content of *L. torquata* (68.50%) was significantly lower than the other two species (*P *<* *0.05, Table 1). The lipid content varied from <5 to >9% across all species (Table 1). A one‐way ANOVA with post hoc tests revealed that *L. torquata* has a significantly higher percentage of lipids in the fresh weight compared to *L. undulata* (*P *<* *0.05), but neither of these species were significantly different to *T. militaris* (Table 1). Conversely, analysis of protein found that *T. militaris* had significantly lower proportions than those of *L. torquata* (*P *<* *0.05) and *L. undulata* (*P *<* *0.05). Protein constituted between 16 and 20% w/w protein of the flesh (Table 1). The carbohydrate content was estimated indirectly as the remaining percent weight at around 3% w/w of the flesh and showed no significant differences between species (Table 1).

**Table 1:** Proximate analysis of the foot tissue from three different turban snails

Species	Proximate contents (%)				
	Ash	Moisture	Lipid	Protein	Carbohydrate
*L. torquata* a	2.10 ± 0.14	68.50 ± 0.64^a^	8.46 ± 0.52^a^	18.03 ± 0.41^a^	2.92 ± 0.50
*L. undulata*	1.97 ± 0.11	70.83 ± 0.95^b^	5.20 ± 0.61^b^	18.49 ± 0.47^a^	3.51 ± 0.53
*T. militaris* a	2.14 ± 0.05	73.08 ± 1.15^b^	5.57 ± 1.07^ab^	16.19 ± 0.11^b^	3.02 ± 0.52

Significant differences between species (*P *<* *0.05) are indicated by different superscript letters in the same column.

a Means and ± SEs from 4 replicates (except *L. undulata* = 6 replicates).

### Fatty acids analysis

**Figure 2:**
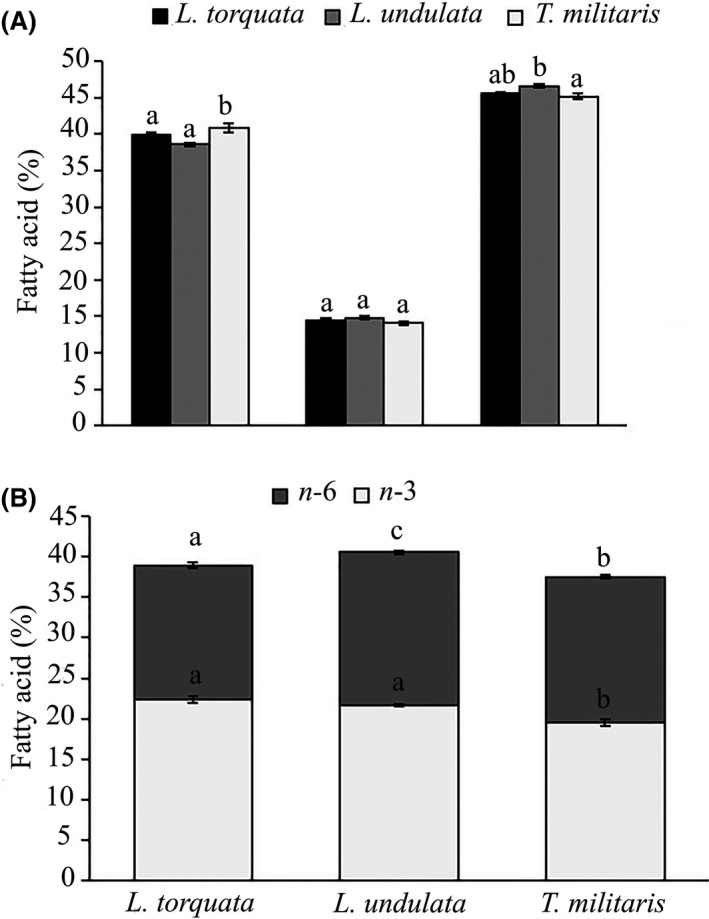
The composition of fatty acids for the three turbinid species with A) the proportion of saturated fatty acid; SFA, monounsaturated fatty acid; MUFA, and polyunsaturated fatty acid; PUFA and B) the proportion of n‐3 and n‐6 fatty acids. Results are presented as mean ± SE. Different superscripts above indicate significant difference between species, with separate univariate PERMANOVAs performed for each class of fatty acid (*P *< 0.05).

The fatty acid composition of the lipid extracts from the foot tissue of the three species is presented in Table 2. Fatty acid profiles show a high proportion (>45%) of polyunsaturated fatty acids (PUFA) and saturated fatty acids (SFA) (>39%), and only ~14% monounsaturated fatty acids (MUFA) (Fig. 2A). The mean ratio of saturated to unsaturated fatty acids is 0.63 for *L. undulata*, 0.65 for *L. torquata,* and 0.69 for *T. militaris*. Univariate PERMANOVA revealed no significant difference between species in the percentage of MUFA (*F* = 2.26, *P *>* *0.05). However, there was a significant difference between species in the percentage of SFA (*F* = 5.73, *P *<* *0.05) and PUFA (*F* = 5.35, *P *<* *0.05). Pairwise analyses revealed a significantly higher proportion of SFA in *T. militaris* compared to *L. torquata* (*P *<* *0.05) and *L. undulata* (*P *<* *0.05), but there was no significant difference in SFAs between *L. undulata* and *L. torquata* (*P *>* *0.05). Pairwise analyses in the percentage of PUFA showed a significantly higher proportion of PUFA in *L. undulata* compared to *T. militaris* (*P *<* *0.05), but neither of these species was significantly different to *L. torquata* (*P *>* *0.05).

**Table 2:** The fatty acid compositions of three turban snails (% of total fatty acids)

Fatty acid	Trivial name	*L. torquata*(*n *=* *4)	*L. undulata*(*n *=* *6)	*T. militaris*(*n *=* *4)
C14:0	Myristic	0.55 ± 0.05	0.96 ± 0.05	0.13 ± 0.05
C15:0	Pentadecanoic	1.18 ± 0.12	1.08 ± 0.09	1.49 ± 0.10
C16:0	Palmitic	23.01 ± 0.41	21.62 ± 0.41	22.12 ± 0.37
C17:0	Margaric	2.39 ± 0.04	1.87 ± 0.09	2.99 ± 0.10
C18:0	Stearic	5.68 ± 0.14	6.59 ± 0.15	5.45 ± 0.08
C24.0	Lignoceric	7.14 ± 0.33	6.49 ± 0.35	7.65 ± 0.22
C16:1	Palmitoleic	3.24 ± 0.10	2.41 ± 0.14	3.00 ± 0.13
C18:1(*n*−9)	Oleic	8.21 ± 0.22	8.41 ± 0.17	7.89 ± 0.18
C20:1(*n*−9)	Eicosenoic	2.77 ± 0.07	3.56 ± 0.17	2.86 ± 0.12
C22:1(*n*−9)	Erucic	0.23 ± 0.03	0.45 ± 0.06	0.30 ± 0.04
C18:2(*n*−6)(LA)	Linoleic	1.60 ± 0.14	2.82 ± 0.09	2.92 ± 0.23
C18:3(*n*−3)(ALA)	a‐ Linoleic	0.87 ± 0.12	2.33 ± 0.09	1.87 ± 0.20
C20:2	11, 13‐ Eicosadienoic	0.06 ± 0.02	0.21 ± 0.03	0.13 ± 0.02
C20:3(*n*−3)(ETA)	Eicosatrienoic	0.16 ± 0.01	0.43 ± 0.05	0.27 ± 0.05
C20:4(*n*−6)(ARA)	Arachidonic	14.93 ± 0.44	16.01 ± 0.24	15.06 ± 0.25
C20:5(*n*−3)(EPA)	Eicosapentaenoic	5.29 ± 0.25	4.63 ± 0.30	3.70 ± 0.12
C22:2	5, 13‐ Docosadienoic	6.61 ± 0.35	5.83 ± 0.21	7.48 ± 0.24
C22:6(*n*−3)(DHA)	Docosahexaenoic	0.80 ± 0.23	0.53 ± 0.04	0.37 ± 0.02
C22:5(*n*−3)(DPA)	Docosapentaenoic	15.27 ± 0.52	13.79 ± 0.26	13.33 ± 0.10

Values are mean value ± SEs (% of total fatty acids)

In all species, the SFA were dominated by palmitic acid (C16), lignoceric acid (C24), and stearic acid (C18). The MUFA with the highest levels in all the three turban snails was oleic acid (C18:1(*n*−9)) (Table 2). Docosapentaenoic (C22:5(*n*−3)), arachidonic acid (C20:5(*n*−6)), docosadienoic (C22:2(*n*−6)), and eicosapentaenoic (20:5(*n*−3)) were identified as the primary PUFAs (Table 2).

The foot tissues of the three turban snails have a mean *n*−3: *n*−6 ratio of 1.36 for *L. torquata*, 1.15 for *L. undulata,* and 1.09 for *T. militaris* (Fig. 2B). Univariate PERMANOVA revealed a significant difference in the percent of *n*−3 (*F* = 14.16, *P *<* *0.05) and *n*−6 (*F* = 11.45, *P *<* *0.05) fatty acids between species. Pairwise analyses for the *n*−3 fatty acids revealed significantly lower levels in *T. militaris* compared to *L. undulata* (*P *<* *0.05) and *L. torquata* (*P *<* *0.05), whereas *n*−6 fatty acids were significant lower in *L. torquata* compared to *T. militaris* (*P *<* *0.05) and *L. undulata* (*P *<* *0.05) (Fig. 2B).

**Figure 3:**
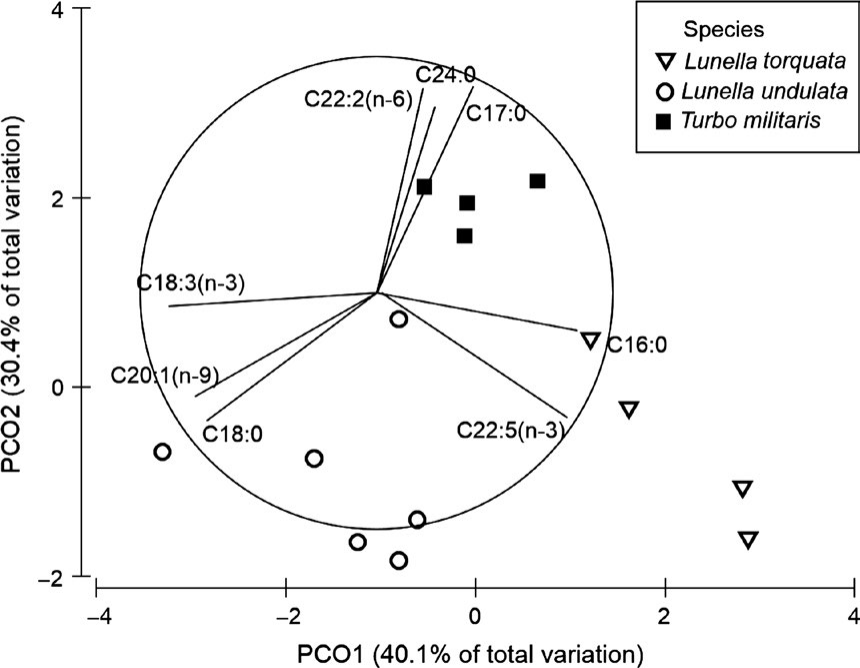
Principle component ordination (PCO) of the fatty acids profiles from the three turban snails based on a Euclidian distance similarity matrix of the percent composition data with vector overlay from Pearsons correlation >0.8.

Multivariate analysis using PERMANOVA revealed that there was a significant difference in the overall fatty acid composition between the three species of turban snails (*F* = 7.39, *P *<* *0.05). Pairwise tests revealed that the composition of fatty acids was different between each pair of species (*P *<* *0.05). A Principal Component Ordination (PCO) was used to explore the differences in the fatty acid profiles of the three turban snails. Separation along the X axis distinguishes *L. torquata* which is characterized by higher docosapentaenoic (C22:5(*n*−3)) and palmitic acids (C16) and *L. undulata* which contains high stearic acid (C18) and a tendency toward high *α* linolenic (C18:3 (*n*−3)) and eicosenoic acid (C20:1(*n*−9)) (Fig. 3). Separated along the Y axis is driven by *T. militaris* which is characterized by higher docosadienoic (C22:2(*n*−6)), lignoceric (C24), and margaric acid (C17:0) (Fig. 3).

### Minerals

The mean concentrations of macronutrients and trace elements found in the foot tissue of the three Turbinidae species are summarized in Table 3. Sodium (Na), potassium (K), calcium (Ca), magnesium (Mg), phosphorus (P), sulfur (S), iron (Fe), copper (Cu), zinc (Zn), molybdenum (Mo), cobalt (Co), selenium (Se), manganese (Mn), aluminum (Al), arsenic (As), cadmium (Cd), chromium (Cr), nickel (Ni), cobalt (Co), lead (Pb), silver (Ag), and mercury (Hg) were assessed in edible foot tissue of the three species. The most abundant mineral was S with the lowest concentration of 8.37 mg/g of the fresh weight found in *T. militaris*. Na and K were also high in all three species, whereas Ca in *L. torquata* was found in higher concentrations than the other two species (Table 3). Among the other elements, *L. undulata* had the highest concentrations of Fe (41.10 ± 3.12 mg/Kg) and Zn (15.20 ± 0.99 mg/Kg).

**Table 3:** Trace elements compositions in three species of turban snails

Elements	*L. torquata*(*n *=* *4)	*L. undulata*(*n *=* *6)	*T. militaris*(*n *=* *4)
Macroelements (mg/g FW)			
Na	3.01 ± 0.12	2.70 ± 0.16	4.00 ± 0.34
K	3.05 ± 0.07	3.33 ± 0.06	2.73 ± 0.08
Ca	2.39 ± 1.11	0.44 ± 0.11	0.61 ± 0.28
Mg	0.69 ± 0.02	0.65 ± 0.03	0.77 ± 0.05
P	1.53 ± 0.09	1.64 ± 0.05	1.22 ± 0.03
S	11.22 ± 0.30	12.32 ± 0.31	8.37 ± 0.18
Microelements (mg/Kg FW)			
Fe	32.42 ± 6.93	41.10 ± 3.12	19.32 ± 1.04
Zn	14.01 ± 1.03	15.20 ± 0.99	12.21 ± 0.89
Cu	1.14 ± 0.12	0.55 ± 0.11	2.18 ± 0.29
Mo	0.15 ± 0.08	0.07 ± 0.004	0.10 ± 0.01
Co	0.03 ± 0.01	0.05 ± 0.01	0.02 ± 0.00
Se	0.177 ± 0.06	0.144 ± 0.02	0.177 ± 0.04
Toxic elements (mg/Kg FW)			
Mn	0.49 ± 0.08	0.45 ± 0.07	0.49 ± 0.14
Al	8.73 ± 4.80	11.12 ± 2.29	0
As	8.84 ± 3.69	5.03 ± 0.53	8.52 ± 1.44
Cd	0.06 ± 0.01	0.05 ± 0.004	0.04 ± 0.01
Cr	0.46 ± 0.33	0.10 ± 0.03	0.17 ± 0.01
Ni	0.05 ± 0.03	0.06 ± 0.02	0.14 ± 0.02
Pb	1.09 ± 0.19	1.04 ± 0.15	2.05 ± 0.62
Ag	0.15 ± 0.03	0.17 ± 0.05	0.04 ± 0.01
Hg	0.007 ± 0.004	0.004 ± 0.002	0.001 ± 0.001

All data presented based on fresh weight. Values are mean value and ± SE: n, number of samples.

**Figure 4:**
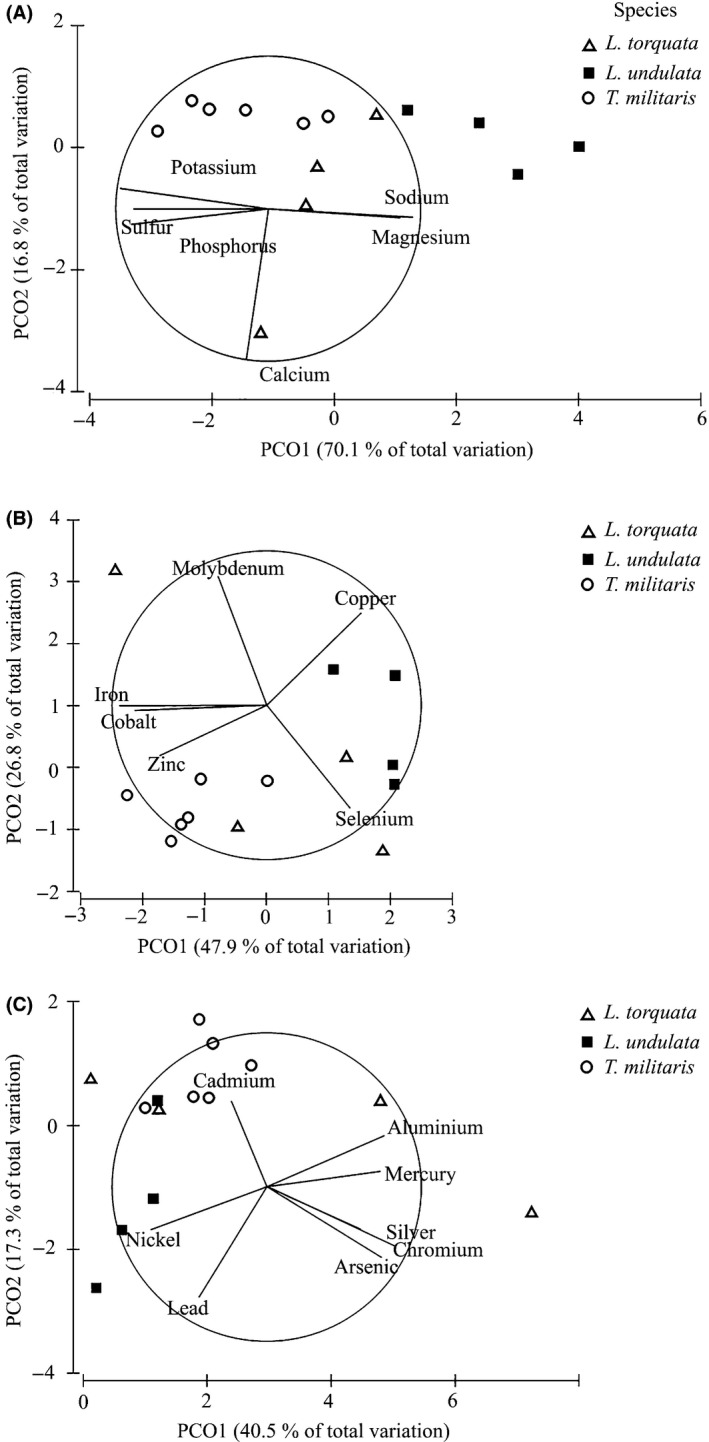
PCO plotted for (A) macro element, (B) microelement, and (C) toxic elements composition of the three turban snails based on a Euclidian distance similarity matrix of the percent composition data with vector overlay from Pearson's correlation >0.5.

PERMANOVA analysis on the normalized macroelemental composition revealed a significant difference between the three species (*F* = 9.38, *P *<* *0.05). Pairwise tests confirmed that the composition of macroelements was different between all pairs of species (*P *<* *0.05). The PCO plot illustrates the difference in macronutrient composition between the three species (Fig. 4A). There is greater variation between individuals of *L. torquata* and *T. militaris*, whereas *L. undulata* form a relatively tight cluster of points characterized by higher sulfur, potassium, and phosphorus. The X axis is largely driven by one outlier of *T*. militaris, which is characterized by higher sodium and magnesium (Fig. 4A).

For the microelements, PERMANOVA analysis found a significant difference between species (*F* = 3.34, *P *<* *0.05). Pairwise analyses revealed significantly different microelement concentrations only between *L. undulata* and *T. militaris* (*P *<* *0.05), whereas *L. torquata* was not different to either *T. militaris* (*P *>* *0.05) or *L. undulata* (*P *>* *0.05). PCO of the microelements (Fig. 4B) reveals a similar pattern as observed for the macroelements (Fig. 4B). *L. undulata* tends to have a relatively tight cluster of samples with relatively more zinc compared to the other species (Fig. 4B, Table 3), whereas *T. militaris* shows a tendency toward higher copper and relatively less Zn than *L. undulata*. Samples of *L. torquata* are interspersed across the full range of the other two species, consistent with no significant differences in the micronutrient composition.

PERMANOVA analysis identified a significant difference between three species (*F* = 2.46, *P *<* *0.05) for trace heavy metals. However, pairwise comparison confirmed there was a significant difference only between *T. militaris* and *L. undulata* (*P *<* *0.05), whereas *L. torquata* was not different to either *T. militaris* (*P *>* *0.05) or *L. undulata* (*P *>* *0.05). PCO was used to explore the differences in the heavy metal composition between the three species (Fig. 4 C). Tighter clustering in *L. undulata* with higher cadmium, appears to explain the difference between *L. undulata* and *T. militaris*, whereas *T. militaris* tends to have higher nickel. The X axis is driven by one outlier of *L. torquata* which characterized by higher chromium, silver, and arsenic.

## Discussion

Evaluation of the nutritional content of three Australian species in the Turbinidae family of gastropod molluscs indicates that these turban snails have good nutritional value, comparable to other shellfish from the other parts of the world. The main outcomes of this study are consistent with previous research on marine molluscs showing high protein (Linehan et al. 1999; Dridi et al. 2007; Periyasamy et al. 2011), as well as low lipid content, which is high in essential fatty acids (Nichols et al. 1998; Chan et al. 2004; Laxmilatha 2009) and a rich source of essential minerals such as zinc (Zn) and iron (Fe). Overall, this study supports the nutritional value of these Australian Turbinidae species and inclusion as part of a healthful human diet.

Protein plays a vital role in molluscs as it serves as an energy supply (Mao et al. 2006). The range of protein content in this study of 16% to 19% of the fresh weight is comparable to other herbivorous gastropods, for example, abalone (Haliotidae), which were reported to have 18.0 ± 0.7% (Chiou et al. 2001) and the turbinid *Cookia sulcata* which was found to contain 17.5 ± 1.5% of protein in the foot tissue (Mason et al. 2014). By comparison, studies on predatory gastropod whelks have generally found higher protein than that reported for herbivores, for example, the Muricidae *Chicoreus ramosus* 36% (Xavier Ramesh and Ayyakkannu 1992); *Hexaplex trunculus* 48% (Zarai et al. 2011); *Rapana venosa* 55.88 ± 2.04% (Celik et al. 2014) and as high as 80% in *Thais haemastoma* (Belisle and Stickle 1978). *Babylonia spirata*, which belongs to another family of predatory and scavenging whelks (Babyloniidae), was reported to exhibit a similar range of protein (53.86%), as found in Muricidae whelks (Periyasamy et al. 2011). Although the trophic niche appears to influence the protein content in gastropod molluscs, with higher contents in predators, the level of protein detected in Turbinidae in this study still suggests that these herbivorous snails are a very good potential source of protein. The protein content can vary depending on the organism, different body parts and seasonal variations (Smoothey 2013). In this study, only the foot tissue was sampled and all species were collected from the same place at the same time of year for standardized comparison. Future studies on the spatial and temporal changes of biochemical composition over the reproductive cycle are therefore recommended.

After proteins, lipids were the next most dominant organic component of the Turbinidae flesh. According to previous findings, the foot tissue of gastropods commonly has less than 10% w/w lipid content (Mclachlan and Lombard 1980; Chiou et al. 2001; Ramesh and Ravichandran 2008; Saito and Aono 2014). The lipid content often appears to be lower (0.5–5% w/w) in predatory gastropods (Belisle and Stickle 1978; Xavier Ramesh and Ayyakkannu 1992; Vasconcelos et al. 2009; Merdzhanova et al. 2014), in comparison to the herbivorous Turbinidae (5–9% w/w, Table 1). Similarly, herbivorous abalone *Haliotis tuberculata* were found to contain up to 6.46% lipid in their foot tissue (Hernandez et al. 2013). Lipid content in the viscera of gastropods appears to be higher than the foot tissue, ranging from 15 to 17% (Chiou et al. 2001). It has been suggested that the visceral tissue is the main storage of site for lipids in gastropods (Dunstan et al. 1996; Saito and Aono 2014). However, in this study we only focused on the foot tissue, since the viscera are not typically consumed by people in larger gastropods. Overall these findings confirm that the foot tissue of gastropods is suitable for inclusion in a high protein, low fat human diet.

This study presents the fatty acid compositions of the foot tissue of three edible Turbinidae *L. torquata*,* T. militaris,* and *L. undulata* from New South Wales, Australia. Palmitic acid (C16:0) is the dominant saturated fatty acid found in all three turban species. This finding is in agreement with previous studies on other molluscs, for example, *Haliotis fulgens*, the pulmonate land snail *Helix aspersa maxima* and the oyster *Crassostrea rhizophorae* (Nelson et al. 2002; Milinsk et al. 2003; Martino and Cruz 2004). According to Gabbott (1983), palmitic acid is the major end‐product of fatty acid synthesis in animal tissue and is the precursor for *de novo* synthesis of long‐chain saturated and unsaturated fatty acids. It is not surprising that Turbinidae have a high proportion of palmitic acid since they mainly feed on macroalgae, which have also been found to contain a high proportion of C16 saturated acid (Freiji and Awadh 2010; Saito and Aono 2014).

Other dominant fatty acids found in the Turbinidae (C18:1(*n*−9): oleic acid, C20:4(*n*−6): ARA, and C22:5(*n*−3): DPA) are very similar to those previously reported from other gastropods, including *Haliotis laevigata*,* Haliotis rubra,* and *Turbo cornutus* (Dunstan et al. 1996; Saito and Aono 2014). In contrast, previous studies on the lipid composition of bivalves (e.g., *Donax deltoides*,* Dosinia caerulea,* and *Perna canaliculus*) indicates that bivalves have relatively higher percentages of EPA and DHA, as opposed to ARA and DPA (Nichols et al. 1998; Mooney et al. 2002). Essential fatty acids such as EPA, DHA, and ARA, cannot be synthesized by molluscs and must be obtained from their food intake. Due to this, the quality and quantity of the food is likely to be the main source of variation in these PUFAs from molluscan tissue. Turban snails are generalist herbivores that feed on a variety of macroalgae (Foster and Hodgson 1998; Davis et al. 2005). Consequently, subtle differences in the macroalgal diet of the three species could explain the significant differences in fatty acid composition between these species collected from the same location.

Several studies comparing the fatty acid composition of marine algae have demonstrated differences between green, red, and brown algae. For example, the green macroalgae, *Caulerpa* sp. was found to be dominated by shorter chain trienoic acids including C16:3 and C18:3 (ALA), whereas the brown algae *Cladosiphon* sp. contained C18 and C20 PUFAs including C18:4(*n*−3), ARA, and EPA (Saito and Marty 2010). C16:3 was also found in the green alga *Enteromorpha*, but not in *Ulva* or several species of red and brown algae (Ragonese et al. 2014). A study on the dietary preferences of *Lunella undulatus* found that they will only consume low levels of *Caulerpa* when offered no choice and much prefer to feed on less chemically defended algae, such as *Ulva* and brown foliose algae (Davis et al. 2005). Furthermore, *L. torquata* feeding was significantly deterred by *Caulpera* extracts (Davis et al. 2005). This is consistent with absence of C16:3 in the turban snails. On the other hand, small amounts of DPA were detected in *Caulerpa* (Saito and Marty 2010), but not in a range of other algae (Ragonese et al. 2014). The turban snails have relatively, high proportions of DPA (>13%) compared to all algae (<1%), but lower proportions of DHA (<1%). DHA was primarily detected in brown algae (1–2%), but was absent in the green algae, including *Ulva* (Ragonese et al. 2014). A number of reports have confirmed the small DHA amount in other gastropod species such as *Littorina littorea* (Ackman and Hooper 1973), *Haliotis laevigata* and *Haliotis rubra* (Dunstan et al. 1996). A seasonal study on adult green abalone, *Haliotis fulgens* found that they have higher DPA in foot tissue when fed the chlorophyte *Ulva lobata* (8.3–12.2%), compared to those fed on the phaeophyte *Egregia menziesii* (5.7–11.8%) or the rhodophyte *Chondracanthus canaliculatus* (7.9–10.9%) (Nelson et al. 2002). These findings confirm that the macroalgae consumed by herbivorous gastropods can influence the overall fatty acid composition and may explain some of the differences between individuals and species. Other biotic and environmental factors such as reproductive status, salinity, temperature, and food availability can also influence the fatty acid compositions of marine organisms (Milinsk et al. 2003; Ozogul et al. 2005).

Among the fatty acids, long‐chain PUFAs such as EPA, DHA, DPA, and ARA are found to have health, as well as nutritional benefits to humans, since they are important for reducing cholesterol levels and coronary heart disease, as well as helping to prevent arteriosclerosis and inflammation (Mahaffey 2004; Mahaffey et al. 2008). These PUFAs have been previously reported from a range of marine molluscs including *Ruditapes decussatus*,* Haliotis asinina*,* Unio terminalis*,* Patella depressa*,* Crassostrea gigas*,* Pinctada fucata martensii*,* Turbo cornutus,* and *Ifremeria nautilei* (Morais et al. 2003; Perez‐Comacho et al. 2003; Saito 2004; Ersoy and Sereflisan 2010; Freiji and Awadh 2010; Saito and Hashimoto 2010, 2010; Bautista‐Teruel et al. 2011). In this study, the levels of DHA in foot tissue of turban snails was relatively low, but high proportions of EPA, DPA, and the *n*−6 fatty acid ARA were detected. DHA was also found to be relatively low in abalone tissue (*Haliotis fulgens*) (Nelson et al. 2002). Another study confirmed that abalone (*Haliotis rubra* and *Haliotis laevigata*), have higher DPA, similar to the turbinids, which could due to the capability of the herbivorous gastropods to retrospectively convert DHA to DPA (Dunstan et al. 1996).

The ratio of *n*−3 to *n*−6 fatty acids is a good indicator for measuring the nutritional value of fatty foods. Based on the Department of Health (UK) guidelines, the maximum recommended ratio of *n*−3 to *n*−6 is 4 (Milinsk et al. 2003). In this study, the ratio of *n*−3 to *n*−6 in the three Turbinidae species was less than 1, which is a very healthful ratio, due to high quantities of ARA and DPA. This is consistent with a previous study on *L. undulata*, which reported a ratio of *n*−3 to *n*−6 = 0.9 (Mooney et al. 2002). By comparison, a study on *Turbo coronatus* from Bahrain reported that the ratio of *n*−3 to *n*−6 was almost 2 (Freiji and Awadh 2010), whereas Australian *Haliotis* spp. range from 1.2 to 1.7 (Mooney et al. 2002). There is much evidence that the early human ancestors consumed diets with *n*−3 to *n*−6 ratio 1:1 (Simopoulos 2002), but this ratio has changed considerably in recent centuries with a bias toward more *n*−6 (Martino and Cruz 2004). The imbalance of *n*−3 to *n*−6 ratio has been linked to a large number of diseases, such as cardiovascular disorders and cancer (Martino and Cruz 2004; Freiji and Awadh 2010). This study reveals that turban snails have potential to contribute to a healthful diet by providing a good source of long‐chain PUFA, particularly DPA and ARA.

This study also investigated the ash content and inorganic elements from the Turbinidae flesh. The flesh was found to contain approximately 2% ash w/w. In a previous study on Haliotidae, the ash content of the abalone muscle was similar (1.8%) to Turbinidae, although a higher ash content was reported from the viscera tissue, with 2.8% w/w. Another study on the predatory gastropod *Dicathais orbita* revealed that the snails fed on different diets had different ash content (ranging from 3.5 to 8%) (Woodcock and Benkendorff 2008), thus suggesting that the percentage of ash could be influenced by the proportion of inorganic materials bioaccumulated at higher trophic levels. However, unlike the lipid composition, we found no significant difference in the total ash content of the three Turbinidae species (Table 1), despite the fact that they may have been feeding on different algae. Nevertheless, we did find interspecies differences in the mineral concentrations.

Molluscs have been considered as a good bioindicators for minerals and trace element availability in the surrounding environment (Cravo and Bebianno 2005). The most abundant macroelements in the turban snail's foot tissue were sulfur (S), potassium (K), and sodium (Na). These results are consistent with those of other studies on another turbinid *C. sulcata* as well as the land snail, *Helix pomatia* (Ozogul et al. 2005; Mason et al. 2014) with ranges of S, K, and Na of 5.5–6.0 mg/g, 0.8–3.0 mg/g, and 0.9–4.1 mg/g, respectively. However, sulfur was much higher in our study on east coast Australian turbinids (8–13 mg/g), when compared with *C. sulcata* from New Zealand (Mason et al. 2014). Sulfur is a common dominant inorganic mineral found in seawater and is one of the most essential elements present in animals, as it is incorporated into amino acids, proteins, enzymes, vitamins, and other biomolecules (Komarnisky et al. 2003; Waska et al. 2008). There is no maximum recommendation for sulfur intake in humans (Komarnisky et al. 2003). Turban snails also have relatively high concentrations of sodium (Na) (up to 4 mg/g) tissue, but a 100 g meal of any of these species remains well under the recommended upper level intake for humans, which is 2.3 g per day (National Health and Medical Research Council, 2006). The recommended daily intake (RDI) for potassium (K) is 3 g a day (National Health and Medical Research Council, 2006), which is corresponds to 1000 g of turban snails meat. Turban snails are not a very rich source of K but they appear to be a very good source of other microelements, which are considered essential minerals for human consumption. Iron (Fe) is among the major minerals found in all species and serves vital functions, such as carrying oxygen in hemoglobin of vertebrates (Erkan 2011). This oxygen carrying role is performed by copper (Cu) in hemocyanin in marine molluscs (Bryan et al. 1977; Stoeva et al. 1997), resulting in relatively high copper in the microelement composition. The turbinids in this study seem to have comparable Fe and Cu content to *Haliotis rubra* from southern Australian waters, which contain 7–31 mg/kg of Fe and 0.4–2.4 mg/kg of Cu (Skinner et al. 2004). All these gastropods fall under the upper limit of Fe and Cu uptake for humans, which is 45 mg/day for Fe and up to 10 mg/day for Cu (National Health and Medical Research Council, 2006) (WHO, 2003).

Zn is another important element with multiple biochemical functions in humans (Scherz and Kirchhoff 2006). For prevention of diseases, the recommended tolerable upper intake for Zn is 40 mg/day (National Health and Medical Research Council, 2006). The turban snails in this study contain up to 16 mg/kg of Zn in their tissue suggesting a 100 g meal of these snails could contribute up to 4% the daily required Zn intake. Previous studies have found a variable range in the Zn content of other molluscs, for example cockles 8.06 mg/kg and mussels 21.2 mg/kg (Guerin et al. 2011). In a previous study on abalone, Zn concentration was found to correlate with concentrations in algae and water; as the Zn increased in the algae, Zn also increased in water and abalone (Lin and Liao 1999). This further supports the idea that the level of microelements in an organism reflects the type of food that they feed on (Jakimska et al. 2011).

As a risk assessment, the Joint FAO/WHO Expert Committee on Food Additives (JECFA) have developed safe limit guidelines for chemicals, including heavy metals in human food, based on the recommendations by the World Health Organization (Herrman and Younes 1999). These limits are represented as provisional tolerable weekly intakes (PTWI) (Table 4) to indicate the maximum safe levels an individual can consume each week per kilogram of body weight (Mamtani et al. 2011). Among the nonessential minerals, strikingly elevated arsenic (As) concentrations were found in the foot tissues of three species of turban snails, ranging from 5 to 9 mg/kg. Arsenic is relatively common in the marine environment and can often be concentrated in seafood (Moreda‐Pineiro et al. 2012). However, Arsenic can exist in two forms: organic and inorganic (Ruttens et al. 2012; Mason et al. 2014), with the organic forms of relatively low toxicity, whereas inorganic forms present the greater hazard (Edmonds and Francesconi 1993). The Australian New Zealand food standard recommends the inorganic As concentration should be below a maximum of 1 mg/kg (FSANZ, 2004) (Table 4). In this study, we only measured total As levels and these ranged from 5 mg/kg in *L. undulata* to 8.8 mg/kg in *L. torquata*. In other gastropods, such as abalone, the inorganic As proportion constituted a maximum 1% of total As (Fabris et al. 2006) and a similar proportion was reported by Sloth et al. (2005) after analyzing a variety of seafood samples including fish, bivalves, and crustaceans. Assuming a similar ratio of less than 1% of the total arsenic is inorganic in Turbinidae flesh, the levels found in this study are not likely to constitute a risk to human health.

**Table 4:** The maximum permitted levels of toxic elements in seafood and their provisional tolerable weekly intake (PTWI) based on the Australian New Zealand standards

Toxic element	Maximum Limit (mg/kg)	Reference	PTWI (mg/kg) body weight	Reference
Al	NA		2.0	(FSANZ, 2011)
As (inorganic)	1.0	(Abbott et al. 2003; FSANZ, 2004)	NA	(FSANZ, 2011)
Cd	2.0	(FSANZ, 2004)	0.03	(FSANZ, 2011)
Cr	NA		0.2	(FSANZ, 2013)
Pb	2.0	(Abbott et al. 2003)	0.025	(FSANZ, 2011)
Hg	0.5	(FSANZ, 2004)	0.004–0.005	(Rodellar et al. 2010; FSANZ, 2011)

NA= not applicable.

The highest level of aluminum (Al) concentrations were found in *L. undulata* (11.12 mg/kg) but this element was not detected at all in *T. militaris*. Mason et al. (2014) reported that Al concentrations in the *C. sulcata* ranged from 14.8 to 15.9 mg/kg. In contrast, two bivalve species, *Chamelea gallina* and *Donax trunculus* in Turkey had accumulated Al concentrations that were much higher (Özden et al. 2009). The PTWI for total Al is 2 mg/kg of body weight (Table 4), suggesting the Turbinids should not be consumed regularly in large quantities.

Lead (Pb) is a toxic element that exists in water mostly in particulate form and accumulates in marine organisms (Özden et al. 2009). In this study, Pb concentrations were higher in *T. militaris* (2.05 mg/kg) compared to the other species. This value only just exceeds the maximum range concentration for Australian and New Zealand food safety standard in molluscs of 2 mg/kg (Abbott et al. 2003) (Table 4). Colakoglu et al. (2011) reported that the highest Pb concentrations in the venerid clam from Turkey, *Chamelea gallina* was 3.24 mg/kg, which was higher than this study. As filter feeders, bivalves are more likely to ingest particulate lead than herbivorous gastropods. Average Pb concentration was 0.04 mg/kg in abalone (*H. tuberculata*) (Noel et al. 2011). It is recommended not to consume more than one kilogram of *T. militaris* in a day to maintain a daily lead intake below the recommended level. Gastropod meat rarely constitutes a major portion of people's diets, especially in more developed countries. So the occasional intake of metals above the recommended concentration does not necessarily equate to large intakes over time. In the case of lead the PWTI is 0.025 mg/kg of body weight (FSANZ (2011); Table 4), which is equivalent to 1.75 mg for a 70 kg adult. Serving of 100 g of turban snails could only contribute to 0.2 mg of Pb at the maximum detected level of total Pb of 2.05 mg/kg.

On the other hand, other toxic elements do not exceed the maximum amount as recommended (Table 4). However, similar to biochemical composition, mineral composition of marine foods can vary with seasonal and biological differences (species, size, age, sex, and sexual maturity), geographical area, food availability, and environmental conditions (temperature, contamination) (Cravo and Bebianno 2005; Scherz and Kirchhoff 2006; Kilic and Belivermis 2013; Bilandzic et al. 2014). Consequently, future studies should investigate the temporal and spatial variation in proximate and elemental composition of these Turbinidae from all major fisheries locations.

## Conclusion

This study provides the first comprehensive assessment of the proximate and elemental composition of marine Turbinidae gastropods from NSW Australia. These findings suggest that in general, the three species of turbinids show minor differences in proximate content. However, all three species can be considered as a healthful food source, similar to other accepted molluscs such as abalone. Further research should be carried out to identify factors influencing variation in the proximate composition of the turbinid snails, including effects of the reproductive cycle, age/size, and site‐specific environmental pollution. Further work could help promote consumer acceptance of Turbinidae as a new meat source and value‐add the mollusc fishing industry in countries such as Australia, where relatively few species are consumed by the majority of the population.
